# Clinical Outcomes of Chemotherapeutic Molecules as Single and Multiple Agents in Advanced Non-Small-Cell Lung Carcinoma (NSCLC) Patients

**DOI:** 10.3390/medicina57111252

**Published:** 2021-11-16

**Authors:** Ting Yoon Kwan, Ezharul Hoque Chowdhury

**Affiliations:** Jeffrey Cheah School of Medicine and Health Sciences, Monash University Malaysia, Jalan Lagoon Selatan, Bandar Sunway, Subang Jaya 47500, Selangor, Malaysia; tkwa0002@student.monash.edu

**Keywords:** advanced non-small-cell lung carcinoma, chemotherapy, immunotherapy, clinical outcomes, objective response rate, overall survival

## Abstract

*Background and Objectives*: Lung cancer is the second most common cancer in the world. Non-small-cell lung carcinoma (NSCLC) makes up 85% of all lung cancer cases and the majority of patients are diagnosed when the cancer is advanced. Over the years, many anticancer drugs have been designed and introduced into the market to treat patients with advanced NSCLC. This review aims to discuss the comparative therapeutic benefits of conventional chemotherapeutics and other drugs available for treating advanced NSCLC. *Materials and Methods:* A literature search for first-line treatment of advanced NSCLC was carried out on PubMed and Google Scholar. Objective response rate (ORR) and overall survival were chosen as target endpoints. *Results:* Monotherapy showed lower treatment endpoints compared to combination therapy. Different combinations of platinum-based doublets demonstrated similar efficacies in treating NSCLC. However, pemetrexed–platinum doublets showed significantly better treatment endpoint in patients with non-squamous NSCLC. Most studies showing the best complete response rate (CRR) utilized epidermal growth factor receptor (*EGFR)* tyrosine kinase inhibitors (*TKI*), while most studies producing the best overall survival included programmed death-1/programmed death-ligand 1 (*PD-1*/*PD-L1)* inhibitors in their treatment regimens. *Conclusions:* The findings of this review indicate that targeted therapy using specific inhibitors is now the most promising first-line anticancer treatment available in the market. However, chemotherapy is still effective in treating advanced NSCLC and is viable as a first-line treatment.

## 1. Introduction

Lung cancer is the second most common cancer in the world. It is also the cancer with the highest mortality rate for both sexes combined. In 2020, an estimated 2.2 million new cases of lung cancer were recorded, and it was responsible for the death of 20% of cancer patients around the world [1]. More than half of the patients with lung cancer are diagnosed with advanced lung cancer when they present to doctors with symptoms for the first time and the overall five-year survival rate for lung cancer patients is approximately 20% [2,3,4]. A 2004 World Health Organization (WHO) classification broadly divides lung cancer into small cell lung carcinoma (SCLC) and non-small-cell lung carcinoma (NSCLC) [5]. NSCLC is more common compared to SCLC, with approximately 85% of all lung cancers being NSCLCs [4]. Prognosis of advanced NSCLC is poor and the five-year survival rate of patients with metastasized NSCLC is approximately 7% [3]. Since there is no cure for advanced NSCLC, the best supportive care is often given for palliative purposes. However, most of these patients will not survive longer than a year [6]. Many studies have shown that for patients who are fit to undergo chemotherapy, the treatment improves their prognosis and survival outcomes compared to the best supportive care [7,8,9,10]. Platinum-based anticancer drugs, such as cisplatin, were proven to be beneficial in the treatment of NSCLC patients in the late 1990s and have since become the first-line treatment of advanced NSCLC [10,11]. With the addition of third generation cytotoxic drugs, such as gemcitabine and paclitaxel, newer antifolate drugs (pemetrexed) and anti-vascular endothelial growth factors (*VEGF*), such as bevacizumab, into the chemotherapeutic regimens of patients with advanced NSCLC, this treatment modality has undergone substantial changes over the years, all of which have demonstrated improvement in survivability of patients [11]. In addition to chemotherapy, the discovery of genetic mutations in certain subsets of NSCLC and advancement in the understanding of tumor immunology have enabled the introduction of personalized and targeted therapies into the first-line regimens in treating a specific subset of NSCLC tumors [11,12]. 

Different drugs work differently in treating the cancer. Platinum-based anticancer drugs, such as cisplatin, carboplatin and nedaplatin, work by binding to the DNA of the tumor cells and cause apoptosis [13,14]. Similar to platinum-based drugs, gemcitabine binds to target sites on the DNA of target cells, resulting in cell apoptosis [15]. Paclitaxel and docetaxel bind to microtubules, stabilizing them and subsequently stopping mitotic cell division of cancer cells [16,17]. Vinorelbine is an antimicrotubule agent that causes arrest of cell division at metaphase [18]. Pemetrexed is an antifolate drug that inhibits folate metabolism and purine biosynthesis [19]. Bevacizumab shrinks tumors by reducing the binding of circulatory *VEGF* to the receptors of the tumor cells and therefore reducing the formation of new blood vessels and blood supply to the tumor [20]. Anti-programmed death-1 (anti-*PD-1,* pembrolizumab or nivolumab) inhibits programmed death-1 (*PD-1*) receptors on cytotoxic T-cells and anti-programmed death-ligand 1(anti-*PD-L1*, atezolizumab) prevents the binding of programmed death-ligand 1 (*PD-L1*) to the *PD-1* receptor, downregulating the activation of T-cells and impairing the body’s antitumor immune response [21]. In tumors with positive epidermal growth factor receptor (*EGFR*) mutation, *EGFR* tyrosine kinase (*TK*) inhibitors (gefitinib, erlotinib, afatinib and osimertinib) specifically target and inhibit these mutated enzymes, leading to the activation of anti-apoptotic pathways in the cancerous cells [22,23,24]. Crizotinib is useful in treating tumors showing positive anaplastic lymphoma kinase (*ALK*) translocation and *ROS* proto-oncogene 1 (*ROS1*) rearrangement by inhibiting the tyrosine kinase domain of the mutated *ALK* and the *ROS1* receptor [25].

This study aims to summarize the clinical outcomes of various commercially available anticancer drugs for advanced NSCLC and to compare the efficacy of these common or popular first-line regimens in palliative treatment of the malignancy.

## 2. Materials and Methods

A literature search for “first line treatment for patients with advanced NSCLC” was performed using PubMed and Google Scholar over a period of 12 days (26 February–9 March 2021). The target population in the studies was to have locally advanced (stage 3B) or metastasized (stage 4) NSCLCs that were previously untreated. The list of articles obtained was screened for relevance and the full texts of relevant articles were acquired. Objective response rate (ORR) and overall survival (OS) were chosen as the target endpoints. Responses were recorded as “complete response” (CR), “partial response” (PR), “stable disease” (SD) and “progressive disease” (PD). OS measures the length of time that the patients remained alive since the beginning of their treatments and was represented by median overall survival or median survival time (MST). Time was to be recorded in either units of months (m) or weeks (w). The hazard ratio of death (HR) was to be included if it was available from the study. “Versus” (vs.) would be used to indicate a comparison between different treatment groups. The study population’s age and performance score (PS) were also considered when reviewing the articles. Data that were unavailable would be indicated as “Not Relevant” (NR). Ten articles with the highest complete response rate (CRR) and overall survival, respectively, were picked and are discussed separately in dedicated sections. 

## 3. Results

### 3.1. Monotherapy vs. Cisplatin/Carboplatin-Based Combination Chemotherapy

Studies comparing cisplatin/carboplatin-based chemotherapy with monotherapy of a common chemotherapy drug are included in Table 1. The ORR of the monotherapy arm ranged from 10.5% to 16%, while the ORR of the platinum-based doublet arm ranged from 19% to 43%. For OS, the range of median OS in the monotherapy arm was 5.3–9.3 m and the median OS in combination chemotherapy arm was 6–9.3 m.

### 3.2. Comparison between Dual-Agent Chemotherapy

Table 2 shows 17 studies comparing platinum-based doublet, non-platinum-based doublet and platinum-pemetrexed doublet treatments. The ORR ranged from 22.9% to 56%, with the highest ORR reported in the nedaplatin–docetaxel doublet arm in the study by Shukuya et al. [32]. The range of OS spanned from 8–20 m. The highest OS was seen in the nedaplatin-based chemotherapy arm in Shan et al.’s study [33].

### 3.3. Doublet Chemotherapy vs. Triplet Chemotherapy

Nine studies have been summarized in Table 3, comparing platinum-based triplet with or without bevacizumab and non-platinum-based triplet with platinum-based and non-platinum-based doublet treatments. The range of ORRs in the “doublet” arms was 20–42% while the range of ORRs in the “triplet” arms was 15–54%. For survivability, the median OS of “doublet” arms ranged from 8.3 m to 17.7 m, whereas the range of median OS in “triplet” arms was 8.1–24.3 m Both the highest ORR and median OS were reported by Zhou et al. [49] in the group receiving carboplatin–paclitaxel–bevacizumab.

### 3.4. PD-1/PD-L1 Inhibitors Regimen vs. Chemotherapy-Only Regimen

Table 4 displays eight studies that compared the efficacy of immune checkpoint inhibitor (PD-1/PD-L1 inhibitors) as monotherapy or in combination with conventional chemotherapy when treating patients with positive *PD-L1* expression tumors. When used as monotherapy and in combination with chemotherapy, the range of ORRs was 26–51.9% and 42.6–58.2% respectively; in terms of median OS, the range was 14.4–30 m and 14.2–22 m, respectively.

### 3.5. EGFR TKI Regimens vs. Chemotherapy-Only Regimen

Table 5 shows eight studies which compared the efficacy of *EGFR TK* inhibitors with conventional chemotherapy in treating patients with positive *EGFR* mutations tumors. Types of *EGFR TK* inhibitors that were included were gefitinib, erlotinib, afatinib and osimertinib. When the inhibitors were used as monotherapy, the range of ORR and OS was 56–80% and 19.3–38.6 m, respectively.

### 3.6. ALK TKI Regimens vs. Chemotherapy-Only Regimen

Table 6 compares crizotinib with conventional chemotherapy or other *ALK TK* inhibitors in treating patients with tumors showing positive *ALK* translocation. The range of ORR in patients treated with monotherapy crizotinib was 74–87.5%. Only Wu et al. [80] reported a median OS of 28.5m in a patient group receiving monotherapy crizotinib.

### 3.7. Treatment for Positive ROS1, BRAF and HER2 Mutations

Table 7 lists the common drugs used in treating *ROS-1* rearrangement, *BRAF* and *HER2* mutation positive tumors. No comparison with conventional chemotherapy was made in any of these studies. The highest ORR (72%) and median OS (51.4 m) were both reported by Shaw et al. [85] when treating *ROS-1* rearrangement positive patients with crizotinib.

## 4. Discussion

Fifty-one phase III randomized controlled trials (RCTs) and eight phase II RCTs were included in this review. Most of these studies used the Eastern Cooperative Oncology Group (ECOG) PS score to assess the baseline performance of patients during enrollment and the commonly implemented range of the score was 0–1/2. Definitions of the scores are listed in Table 8.

World Health Organization (WHO) PS and Karnofsky PS (KPS) were also used in a few studies, but they had a good correlation with ECOG PS in their scores [96,97]. The median age range of patients in these studies was 50–66 years and one study by Smit et al. [92] had recruited patients with a median age of 42 years old.

Some of the common drugs used to treat advanced NSCLC and their dosages that were found in this literature review are listed in Table 9.

Median cycles of treatments administered or the median durations of treatments for common regimens found in this literature review are listed in Table 10.

There were more male than female patients in most of the studies, though not in trials that recruited patients with tumors of specific genetic mutation.

Most studies evaluated tumor shrinkage (objective response) following the Response Evaluation Criteria in Solid Tumours (RECIST) 1.1 tumor response criteria but in a few studies, RECIST 1.0, WHO and South West Oncology Group (SWOG) tumor response criteria were used. RECIST 1.0 had highly agreeable results compared with RECIST 1.1 and WHO, and SWOG criteria was not inferior compared to RECIST criteria in clinical evaluation of tumor shrinkage [99,100]. Overall survival was generally defined as time from randomization in the trial until death due to any cause [101].

Monotherapy with platinum-based drugs or cytotoxic drugs generally showed lower ORRs and median OS compared to combination therapy; the median OS for monotherapy was, on average, 7 m [26,27,28,29,30]. When used as monotherapy, cisplatin had a much lower incidence of hematological toxicity compared to cisplatin–vinorelbine doublet [26]. Similarly, vinorelbine monotherapy had notably lower incidence of hematological and non-hematological side effects compared to vinorelbine–cisplatin doublet [27,29].

Six studies revealed that there was no significant difference in ORR between groups of patients that were given different types of platinum-based doublets, suggesting that all platinum-based doublets had comparable efficacy in treating advanced NSCLC [32,34,35,39,41,48]. There was also no significant difference in median OS between different types of cisplatin or carboplatin-based doublets, and the reported MST was on average less than a year [34,36,37,38,39,40,41,42]. Next, cisplatin was noted to show higher ORR and survival benefit compared to carboplatin [38,102,103,104,105]. Nedaplatin-based doublet had an MST of more than 12 m—a significantly higher MST compared to cisplatin-based doublet. Only one study reported a significantly higher ORR in a nedaplatin group compared to a cisplatin group [32,33,43]. Mixed results were reported from different studies regarding the difference in ORR and median OS between platinum-based and non-platinum-based chemotherapy. However, severe myelosuppression, nausea and vomiting were more commonly seen in the platinum-based regimen compared to the non-platinum-based regimen [45,46,50,105,106]. Common adverse effects observed in patients taking platinum-based doublet are non-hematological side effects, such as nausea, vomiting and alopecia, and hematological adverse effects, such as anemia, neutropenia, leucopenia and thrombocytopenia. Cisplatin-based regimens had a higher incidence of toxicity and significant increased chance of causing severe nausea and vomiting compared to carboplatin-based regimens, while carboplatin-based chemotherapy had a significantly higher chance of causing thrombocytopenia [34,38,39,40,41,44,102,104,105]. Nedaplatin had lower rates of serious nausea and vomiting but a higher incidence of thrombocytopenia and neutropenia compared to cisplatin and carboplatin [32,43,48,107]. Toxicity profiles were generally similar across all conventional cisplatin-based doublets, except for the vinorelbine–cisplatin regimen which had a higher rate of severe anemia, neutropenia, leukopenia, nausea and vomiting [34,39,41,42].

When considering only patients with non-squamous NSCLC, the pemetrexed–platinum doublet showed a significantly better response rate and survival benefit compared to other platinum-based doublets, suggesting that pemetrexed had a superior benefit for this subset of NSCLC patients [35,37,47,108,109]. A pemetrexed monotherapy or pemetrexed–platinum regimen had a significantly lesser incidence of severe hematological adverse effects compared to other platinum-based doublets [30,35,36,37,47]. Platinum-based triplets showed both numerical and statistically significant improvement in ORR and median OS when compared to platinum-based doublets. However, they consistently showed significantly higher rates of hematological toxicity when compared to platinum-based doublets [50,51,52,54]. The addition of bevacizumab to a platinum-based doublet resulted in significantly higher ORR and median OS compared to chemotherapy alone [49,53,55,57]. Adding bevacizumab to a regular platinum-doublet regimen was also shown to significantly increase the risk of bleeding events, neutropenia, hypertension, hyponatremia and headache [49,53,57,61]. A high dose of bevacizumab (15 mg/kg) was associated with a 5% higher rate of serious adverse events compared to low dose bevacizumab (7.5 mg/kg) [55].

Pembrolizumab or atezolizumab generally had statistically significant superior OS and a numerically superior ORR when used as either monotherapy or in combination with platinum-based doublets compared to chemotherapy. Additionally, the higher the level of *PD-L1* expression, the greater the benefits of the drugs received by the patients. The use of *PD-1*/*PD-L1* inhibitor was noted to increase the risk of immune-mediated adverse effects, for example, hyperthyroidism, hypothyroidism, pneumonitis and hepatitis. The incidence of these adverse events was similar when the drugs were used as monotherapy or in combination with platinum-based therapy [59,60,61,64,65,66,67,68].

Patients with positive *EGFR* mutations who received *EGFR* inhibitors monotherapy generally only had numerically improved OS but a statistically superior ORR compared to platinum-based chemotherapy [69,70,71,72,73,74,75,79]. However, targeted therapy did not show better performance than chemotherapy when used on patients without these mutations. Two studies by Herbst et al. [69,70] showed that combining *EGFR TK* inhibitors with chemotherapy did not produce statistically significant higher response rates compared to chemotherapy alone in patients with wild type *EGFR*. Similarly, statistically significant improvement in ORR and numerical improvement in OS was observed when treating *ALK* translocation positive patients with crizotinib compared to platinum-based chemotherapy [80,82]. Monotherapy of crizotinib and dabrafenib for patients with *ROS1* tumors and *BRAF* mutations, respectively, showed convincing ORR and OS but no comparison with mainstream therapy was made [85,86,87]. Some common adverse effects due to the use of *EGFR* inhibitors were diarrhea, rashes and acne. When these drugs were used in combination with chemotherapy, the risk of hematological adverse effects were not increased [69,71,72,73,75]. Crizotinib increased the risk of vision loss, diarrhoea, edema and vomiting when used as monotherapy [80,81,82,110]. Lastly, dabrafenib which was commonly prescribed with trametinib for NSCLC increased the risk of pyrexia, asthenia, nausea, vomiting and decreased appetite [87,89].

### 4.1. Top 10 Regimens with the Best “Complete Response” Rate

**Figure 1:**
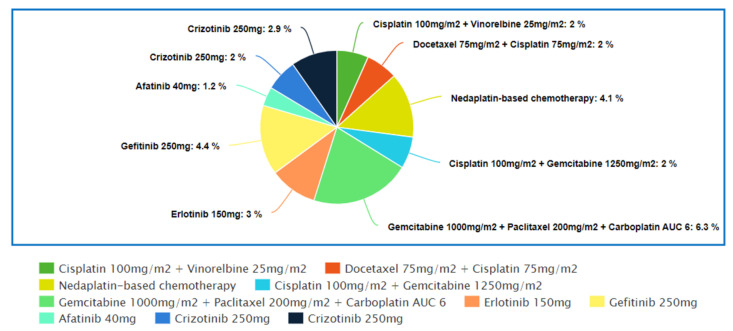
Top 10 regimens with the best “Complete Response” rate.

Figure 1 represents the top 10 regimens that produced the best complete response rate out of all the regimens that were included in this review. The Supplementary Table S1 shows the studies in which these regimens were used.

The trial by Wozniak et al. [26] showed that platinum-based doublet was significantly more effective than platinum-based drug monotherapy. Two percent of patients in the cisplatin–vinorelbine chemotherapy group achieved CR and none in the cisplatin monotherapy group had achieve any CR. However, the incidence of serious hematological adverse effects in the combination regimen group was much higher than the cisplatin monotherapy group, while non-hematological side effects were comparable in both treatment groups. Higher rates of toxicity were also seen in vinorelbine–cisplatin doublet when compared with vinorelbine monotherapy [29]. Despite having higher toxicity rates, patients receiving combination therapy had a median of three cycles of treatment, while the cisplatin monotherapy arm received only two cycles of treatment during the study. Furthermore, the monotherapy arm had twice as many patients with PD than the platinum-doublet arm. Thus, it could be suggested that an increase in treatment efficacy greatly outweighed the increased rate of toxicity in combination chemotherapy.

In a study by Fossella et al. [42], the docetaxel–cisplatin doublet arm had a significantly greater ORR compared to the vinorelbine–cisplatin arm, while the docetaxel–carboplatin doublet arm had a numerically lower but statistically insignificant ORR when compared to the vinorelbine–cisplatin arm. The CR rate for both the cisplatin doublet group was 2% but only 1% for the carboplatin doublet group. This finding was consistent with the results of other studies that showed that cisplatin had an overall greater efficacy compared to carboplatin [38,102,103,104]. The cisplatin–vinorelbine arm had the highest rate of serious toxicity, followed by the docetaxel–cisplatin arm and docetaxel–carboplatin arm. Vinorelbine–cisplatin doublets were also noted to have the highest rate of toxicity in another similar trial comparing cisplatin–vinorelbine doublets with gemcitabine–cisplatin and paclitaxel–carboplatin doublets [34,39,41,111]. The higher rate of toxicity might have caused lower median cycles of treatment received in the vinorelbine–cisplatin arm compared to the docetaxel–cisplatin arm, leading to significantly lower ORRs.

In the study by Alberola et al. [53], the cisplatin–gemcitabine doublet showed a CR rate of 2%, which was consistent with the results from other studies [26,42]. Next, it had a comparable ORR and the same CR rate as the cisplatin–gemcitabine–vinorelbine triplet, but the triplet therapy was associated with a significantly greater incidence of toxicity [54]. In another trial comparing the same triplet and doublet chemotherapy regimen, the triplet chemotherapy also had a numerically higher but statistically insignificant ORR [52]. Alberola et al. [54] also reported that both the doublet and triplet chemotherapy had significantly greater ORRs compared to non-platinum based sequential therapy. Significantly lower rates of hematological toxicity had also been noted in the sequential therapy arm compared to the cisplatin–gemcitabine doublet arm. This trend was consistent with the findings from multiple studies which showed that platinum-based chemotherapy had a greater ORR but worse toxicity, causing, especially, myelosuppression, nausea and vomiting, compared to non-platinum-based chemotherapy [45,46,50]. In one study which compared a cisplatin-based regimen with ifosfamide to an identical regimen without cisplatin, the cisplatin arm had a higher OR (30% vs. 24%) but also a greater rate of serious neutropenia and leukopenia compared to the non-cisplatin arm [112].

Next, Paccagnell et al. [51] reported that the cisplatin–gemcitabine–paclitaxel triplet chemotherapy had shown a statistically significant ORR compared to the paclitaxel–carboplatin doublet (43.6% vs. 20%). The CR rates of the triplet and doublet chemotherapy arm were 6.3% and 0%, respectively. The triplet regimen arm also was assoicated with a significantly greater incidence of anemia, neutropenia and thrombocytopenia compared to the doublet arm [51]. Other studies comparing platinum-based doublets and platinum-based triplets with two cytotoxic drugs yielded an insignificant difference in ORR but a significantly higher rate of hematological adverse events in patients receiving triplet chemotherapy compared to those receiving doublet chemotherapy [50,52,54]. This might suggest that triplet chemotherapy had an ambiguous benefit but conclusively higher toxicities over doublet chemotherapy.

Next, Li et al. [43] reported that a nedaplatin-based doublet had a significantly higher ORR compared to a cisplatin-based doublet. The CR rate achieved by the nedaplatin-based doublet was 4.1% compared to 3.1% by the cisplatin-based doublet. In the subgroup analysis, only the paclitaxel–nedaplatin doublet and gemcitabine–nedaplatin doublet showed a statistically significant increase in ORR when compared to cisplatin–paclitaxel and cisplatin–gemcitabine.

Three trails by Rosell et al. [71], Maemondo et al. [72] and Wu et al. [73] separately studied the efficacy of *EGFR* inhibitors monotherapy compared to platinum-based chemotherapy in treating patients with *EGFR* positive tumors. In these trials, all patients had significantly greater ORRs when given *EGFR TK* inhibitors compared to platinum-based chemotherapy; Gefitinib showed the highest CR rate (4.4%), followed by erlotinib (3.0%) and afatinib (1.2%) [71,72,73]. Three other studies also showed statistically significant improvements in ORR when comparing these *EGFR* inhibitors to chemotherapy [75,78,113]. The ORR of afatinib in Wu et al.’s study [73] was comparable to the ORR of erlotinib in Rosell et al.’s study [71] and the ORR of afatinib in another trial (66.9% vs. 64% vs. 69%) [75]. This finding suggested similar efficiencies between erlotinib and afatinib [114,115]. Common adverse effects seen with the use of *EGFR* inhibitors were diarrhoea, rashes and acne, and the rate of serious adverse effects were comparable among the three studies [71,72,73].

Both trials by Solomon et al. [82] and Wu et al. [80], respectively, had studied the use of crizotinib in the first-line treatment of patients with positive *ALK* translocation NSCLC. In these trials, crizotinib was compared with pemetrexed–cisplatin/carboplatin doublets and yielded statistically significant improvement in ORR. The CR rates observed were 2% and 2.9%, respectively. Vision disorders, diarrhoea, edema and increased transaminase levels were the most reported adverse effects in the crizotinib arms of both trials and were at least 5% more frequent in the crizotinib arm compared to the chemotherapy arm [80,81,82].

### 4.2. Top 10 Regimens with the Best Overall Survival

**Figure 2:**
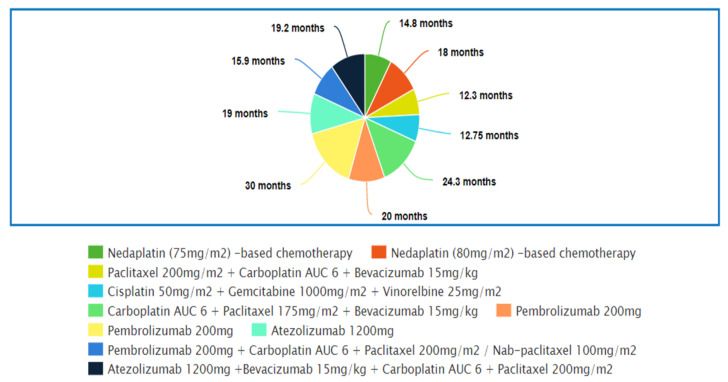
Top 10 regimens with the best Overall Survival (OS).

Figure 2 shows the top 10 regimens that demonstrated the best overall survival. Supplementary Table S2 reveals the studies in which these regimens were used.

In the study by Li et al. [43], a nedaplatin-based regimen showed significantly improved median OS for patients compared to a cisplatin-based regimen. The median OS of the nedaplatin arm was comparable to the median OS of the nedaplatin arm reported in two other trials (13.6 m vs. 14.8 m vs. 17.5 m) which had also shown superior OS compared to cisplatin/carboplatin-based chemotherapy [32,48]. Shan et al. [33] also concluded that nedaplatin-based chemotherapy provided significantly better OS compared to cisplatin-based chemotherapy. Common adverse events seen in the nedaplatin group were increased glutamic-pyruvic transaminase levels, increased creatinine levels and neutropenia. The toxicity profile of nedaplatin-based chemotherapy was generally better; only the rate of elevated glutamic-oxaloacetic transaminase and indirect bilirubin were higher in patients receiving nedaplatin compared to cisplatin [32,33,43,48].

The next trial by Pasquale et al. [52] reported that cisplatin–gemcitabine–vinorelbine triplet chemotherapy provided significantly greater median overall survival compared to cisplatin–vinorelbine doublets, and the difference was 4 m. A statistically significantly longer MST (longer by 2.5 m) in the triplet arm compared to the paclitaxel–carboplatin doublet was noted in another trial [51]. However, two trials comparing triplet to doublets yielded insignificant results [50,54]. In particular, one of the trials that used the cisplatin–gemcitabine–vinorelbine triplet resulted in a numerically lower median survival time when compared to the cisplatin–gemcitabine doublet (8.2 m vs. 9.3 m) [54]. The outcomes of these studies suggested that the survival benefit of triplet over doublet chemotherapy was still uncertain. However, hematological side effects were significantly more common in the triplet chemotherapy arm than in the doublet chemotherapy arm [50,51,52,54].

Sandler et al. [53] and Zhou et al. [49] had compared paclitaxel–carboplatin–bevacizumab triplet with paclitaxel–carboplatin doublet only treatment on non-squamous NSCLC patients in separate trials. Significant improvements were observed in the triplet arm of both trials and the difference in median OS rates were 2 m and 6.6 m, respectively. A meta-analysis reported that the paclitaxel/docetaxel–platinum doublet was shown to have a significant improvement on OS when combined with bevacizumab [116]. The addition of bevacizumab to a cisplatin-based doublet did not increase the risk of hematological adverse effects, and the common adverse effects seen in the bevacizumab arm were hypertension, proteinuria, headaches and WBC count decrease [49,55]. In addition to hypertension, proteinuria and headaches, Sandler et al. [53] reported significantly higher rates of thrombocytopenia and neutropenia in the bevacizumab group. Increased toxicity might explain the higher median cycles of treatment administered in Zhou et al.’s study [49] compared to Sandler et al.’s study (eleven vs. seven) and the difference in median OS of the paclitaxel–carboplatin–bevacizumab group between these two studies (24.3 m vs. 12.3 m).

In the next 5 studies, patients taking pembrolizumab or atezolizumab demonstrated significantly superior median OS compared to patients who only had chemotherapy, and the difference in OS ranged from 4.6 m to 15.8 m [59,61,66,67,68]. Three separate studies from Mok et al. [59], Reck et al. [61] and Giaccone et al. [66] had shown superior survival benefits provided by pembrolizumab or atezolizumab monotherapy compared to platinum-based chemotherapy. Mok et al. [59] and Giaccone et al. [66] also showed that the survival benefit received by patients taking pembrolizumab or atezolizumab increased with the expression of *PD-L1* in the tumor cells. In both studies, the difference in median OS between the control and experimental arms dropped to only 4.6 m and 3.4 m, respectively. However, regardless of the level of expression, significantly greater OS was still observed in the pembrolizumab and atezolizumab arms compared to the chemotherapy arm [59,66]. When comparing the efficacy of pembrolizumab and atezolizumab, comparable median OS was seen in the studies but two meta-analyses reported that the *PD-1* inhibitor had greater efficacy compared to the *PD-L1* inhibitor [59,66,117,118]. Overall, *PD-1*/*PD-L1* monotherapy had a better toxicity profile compared to chemotherapy. The rates of immune-mediated adverse effects, such as hepatitis, rashes, hyperthyroidism and hypothyroidism, were higher in *PD-1*/*PD-L1* inhibitor monotherapy compared to chemotherapy across all three studies. The toxicity profile of pembrolizumab was similar to that of atezolizumab [59,61,66].

When the *PD-1*/*PD-L1* inhibitor was combined with carboplatin-based chemotherapy, both Paz-Ares et al. [67] and Socinski et al. [68] reported significantly longer survival time in the *PD-1*/*PD-L1* inhibitor arm by 4.6 m and 4.5 m, respectively. The median OS reported by Socinski et al. [68] was higher than the median OS reported by Paz-Ares et al. [67] in both arms. This could be explained by the addition of bevacizumab in both arms, as the drug was known to improve the survival outcome of the platinum-based regimen, especially the taxane–platinum chemotherapy regimen [49,53,57,116]. Overall rates of adverse events were higher in groups receiving pembrolizumab/atezolizumab combination therapy compared to groups receiving only chemotherapy. Higher rates of immune-mediated adverse events were observed in the combination therapy arm, while the hematological side effects were similar in both arms [62,65,67,68]. Adverse effects were also significantly more prevalent in *PD-1*/*PD-L1* combination chemotherapy compared to monotherapy [119].

## 5. Limitation

Firstly, the literature search was done only on PubMed and Google Scholar. As a result, some relevant studies might have been unintentionally excluded during the search. Secondly, ten papers that were in this literature review were published before the year 2000. Thirdly, some of the latest regimens or developments in the treatment of NSCLC might not be included in the review because: (1) the studies/guidelines were only published after the completion of the review; (2) of unintentional exclusion of the studies during the literature search; and (3) a regimen or development was still unpopular or insignificant at the time of the completion of this review. For instance, the combination of nivolumab and ipilimumab was approved by the U.S. Food and Drug Administration (FDA) as the first-line therapy for NSCLC with tumors expressing PD-L1 ≥ 1% in May 2020 [120]. Next, the use of tepotinib and capmatinib for NSCLC with the mesenchymal epithelial transition (*MET*) exon 14 skipping mutation was unintentionally missed [121,122]. Finally, entrectinib which was originally being studied for treatment of solid tumors with neutrophic tyrosine receptor kinase (*NTRK*), was approved for the treatment of ROS-1 positive NSCLC [123].

## 6. Conclusions

This review aimed to summarize the clinical studies conducted using commercially available drugs as first-line treatment for patients with advanced NSCLC and compare their efficacy. In addition to the platinum-based chemotherapy, many new drugs that provided significantly greater improvements in tumor response and survival times were introduced into the market. Targeted and personalized therapy using specific inhibitors, such as *EGFR TK* inhibitors, crizotinib and *PD-1*/*PD-L1* inhibitors, should be used as first-line treatment, as these drugs have shown significantly better clinical outcomes with better toxicity profiles compared to standard platinum-based chemotherapy. However, platinum-based chemotherapy with pemetrexed and/or bevacizumab was still effective against NSCLC and should be considered in some cases. In this review, targeted and personalized therapy has shown promising results and should be the emphasis of research and trials in the future, as more knowledge at the biomolecular level of the cancer is continuously evolving.

## Figures and Tables

**Table 1:** Single agent vs. combination chemotherapy.

PS **	Median Age	Regimen	Patients(n)	Objective Response (CR + PR)/SD/PD; ORR (%) *	Median OS/MST ***	Study
0–1	63	Cisplatin monotherapy vs. Cisplatin + Vinorelbine	415	25 (0/25)/72/112; 12% vs. 54 (4/50)/97/55; 26% (*p* = 0.0002)	8 m vs. 6 m (*p* = 0.0018)	1998, Wozniak et al. [26]
WHO PS 0–2	59 vs. 59 vs. 60	Vinorelbine + Cisplatin (NVB-P) vs. Vindesine + Cisplatin (VDS-P) vs. Vinorelbine monotherapy (NVB)	612	57 (NR)/NR/NR; 30% vs.35 (NR)/NR/NR; 19% vs.28 (NR)/NR/NR; 14% NVB-P vs. VDS-P (X^2^, *p* = 0.02) NVB-P vs. NVB (X^2^, *p* < 0.001)	40 w vs. 32 w vs. 31 w NVB-P vs. VDS-P (*p* = 0.4) NVB-P vs. NVB (*p* = 0.1)	1994, Le Chevalier et al. [27]
KPS 70–100	62 vs. 63	Gemcitabine + Cisplatin vs. Cisplatin monotherapy	522	79 (3/76)/97/38; 30.4% vs. 29 (1/28)/111/86; 11.1% (*p* < 0.0001)	9.1 m vs. 7.6 m (log-rank test, *p* = 0.004)	2000, Sandler et al. [28]
WHO PS 0–2	58.8 vs. 59.2	Vinorelbine monotherapy vs. Vinorelbine + Cisplatin	240	18 (1/17)28/58; 16% vs. 50 (2/48)/35/19; 43% (*p* = 0.0001)	32 w vs. 33 w (log-rank test *p* = 0.48)	1994, Depierre et al. [29]
ECOG PS 2	65 vs. 65	Pemetrexed monotherapy vs. Pemetrexed + Carboplatin	217	7; s10.5% vs. 19; 24% (*p* = 0.32)	5.3 (95% CI, 4.1–6.5 m) vs. 9.3 m (95% CI, 7.4–11.2 m) (HR, 0.62; 95% CI, 0.46–0.83, *p =* 0.001)	2013, Zukin et al. [30]

* “Complete Response” (CR): Disappearance of all target lesions; “Partial Response” (PR): 30% or more decrease in the sum of diameter of target lesions compared to baseline diameter; “Stable Disease” (SD): Neither PR nor PD; “Progressive Disease” (PD): 20% or more increase in sum of diameter of target lesions compared to baseline diameter; “Objective response” = CR + PR [31]. ** “Performance Score” (PS): Eastern Cooperative Oncology Group (ECOG) PS, World Health Organization (WHO) PS, Karnofsky PS (KPS). *** “Overall Survival” (OS), “Median Survival Time” (MST).

**Table 2:** Comparison among doublet chemotherapy.

PS **	Median Age	Regimen	Patients (n)	Objective Response (CR+PR)/SD/PD; ORR (%) *	Median OS/MST ***	Study
ECOG PS 0–2	63	Cisplatin + Paclitaxel vs. Cisplatin + Gemcitabine vs. Cisplatin + Docetaxel vs. Carboplatin + Paclitaxel	1155	(<1% + 21%)/18%/49%; 21% vs. 22% (1% + 21%)/18%/40%; 22% vs. (<1% + 17%)/25%/42%; 17% vs. (<1% + 16%)/23%/49%; 17%	7.8 m (95% CI, 7.0–8.9 m) vs. 8.1 m (95% CI, 7.2–9.4 m) vs. 7.4 m (95% CI, 6.6–8.8 m) vs. 8.1 m (95% CI, 7.0–9.5 m)	2002, Schiller et al. [34]
ECOG PS 0–1	61.1 vs. 61.0	Cisplatin + Pemetrexed vs. Cisplatin + Gemcitabine	1725	30.6% vs. 28.2%	10.3 m vs. 10.3 m (HR = 0.94, 95% CI, 0.84–1.05)	2008, Scagliotti et al. [35]
WHO PS 0–2	64 vs. 66	Pemetrexed +Carboplatin vs. Gemcitabine + Carboplatin	446	NR	7.3 m vs. 7.0 m (*p* = 0.63)	2009, Grønberg et al. [36]
ECOG PS 0–2	60.1 vs. 58.9	Pemetrexed +Carboplatin vs. Docetaxel + Carboplatin	260	34% vs. 22.9% (OR = 1.68 (95% CI: 0.91–3.10), *p* = 0.095)	HR = 0.93 (95% CI: 0.66–1.32), *p* = 0.698	2011, Rodrigues-Pereira et al. [37]
KPS 70–100	63 vs. 62	Gemcitabine + Cisplatin vs. Gemcitabine + Carboplatin	176	36 (5 + 31)/29/16; 41.4% (95% CI: 31.0–51.7%) vs. 26 (5 + 21)/39/21; 29.2% (95% CI: 19.8–38.7%) (*p* = 0.09)	8.75 m (95% CI: 6.7–10.5 m) vs. 8 m (95% CI: 6.9–11.4 m) (*p* = 0.9024)	2003, Zatloukal et al. [38]
ECOG PS 0–1	62 vs. 63 vs. 61 vs. 61	Cisplatin + Irinotecan (IP) vs. Paclitaxel + Carboplatin (TC) vs. Cisplatin + Gemcitabine (GP) vs. Cisplatin + Vinorelbine (NP)	602	31% vs. 32.4% (*p* = 0.801 *) vs. 30.1% (*p* = 0.868 *) vs. 33.1% (*p* = 0.706 *) * Compared with IP by the x^2^ test.	13.9 m vs. 12.3 m vs. 14.0 m vs. 11.4 m	2007, Ohe et al. [39]
PS 0–1	61 vs. 62	Cisplatin + Vinorelbine vs. Paclitaxel + Carboplatin	408	56 (0 + 56)/53/56; 28% vs. 52 (2 + 50)/67/53; 25% (*p* = NS)	8.1 m (95% CI, 6.7–9.6 m) vs. 8.6 m (95% CI, 7.2–10.7 m) (*p* = 0.87)	2001, Kelly et al. [40]
ECOG PS 0–2	63 vs.62 vs.63	Gemcitabine + Cisplatin (GC) vs. Paclitaxel + Carboplatin (PCb) vs. Vinorelbine + Cisplatin (VC)	607	62 (0 + 62)/81/36; 30% (95% CI 24–37%) vs. 64 (1 + 63)/75/37; 32% (95% CI 25–38%) vs. 61 (1 + 60)/62/34; 30% (95% CI 24–36%) (GC vs. VC, *p* = 0.982) (PCb vs. VC, *p* = 0.747)	9.8 m (95% CI, 8.6–11.2 m) vs. 10.0 m (95% CI, 9.0–12.5 m) vs. 9.5 m (95% CI, 8.3–11.0 m) * No differences between experimental arm and reference arm (VC)	2002, Scagliotti et al. [41]
KPS 70–100	61 vs. 59 vs. 61	Docetaxel + Cisplatin (DC) vs. Docetaxel + Carboplatin (DCb) vs. Vinorelbine + Cisplatin (VC)	1218 patients	129 (8 + 121)/176/72; 31.6% (95% CI 27.1–36.4%) vs. 97 (5 + 92)/188/88; 23.9% (95% CI 19.8–28.3%) vs. 99 (8 + 91)/170/86; 24.5% (95% CI 20.4–29.0%) DC vs. VC (*p* = 0.029) DCb vs. VC (*p* = 0.870)	DC vs. VC = 11.3 m vs. 10.1 m (HR = 1.183 (97.2% CI, 0.989–1.416)) * Not statistically significant DCb vs. VC = 9.4 m vs. 9.9 m (HR = 1.048 (97.2% CI, 0.877–1.253)) * Not statistically significant	2003, Fossella et al. [42]
ECOG PS 0–1	64 vs. 65	Nedaplatin + Docetaxel vs. Cisplatin + Docetaxel	355	96 (3 + 93)/50/NR); 56% vs. 89 (1 + 88)/47/NR); 53% (two-sided Fisher’s exact test, *p* = 0.66)	13.6 m (95% CI 11–15.6 m) vs. 11.4 m (95% CI 10.2–12.2 m) (HR 0.81, 95% CI 0.65–1.02; one-sided stratified log-rank, *p* = 0.037)	2015, Shukuya et al. [32]
NR	NR	Nedaplatin + Gemcitabine/Paclitaxel/Navelbine/Docetaxel/Cyclophosphamide + Doxorubicin vs. Cisplatin + Gemcitabine/Paclitaxel/Navelbine/Docetaxel/Cyclophosphamide + Doxorubicin	392	NR	20 m (95% CI 17.0–23.0 m) vs. 15 m (95% CI 13.4–16.6 m) (*p* = 0.022)	2015, Shan et al. [33]
NR	56.28 vs. 55.01	Nedaplatin Group (NDP + Pemetrexed/Docetaxel/Gemcitabine/Vinorelbine/Paclitaxel) vs. Cisplatin Group (DDP + Pemetrexed/Docetaxel/Gemcitabine/Vinorelbine/Paclitaxel)	619	143 (12 + 131)/137/14; 48.6% vs. 114 (10 + 104)/176/35; 35.1% (*p* < 0.01)	(14.783 ± 1.092) m vs. (13.502 ± 2.327) m (*p* < 0.01)	2014, Li et al. [43]
ECOG 0–2	58 vs. 58	Paclitaxel + Cisplatin vs. Paclitaxel + Carboplatin	618	80 (2 + 78)/123/58; 28% (95% CI 23–34%) vs. 70 (4 + 66)/112/80; 25% (95% CI 20–31%) * Paclitaxel/Carboplatin is statistically non-inferior compared to paclitaxel/cisplatin	9.8 m (95% CI 8.2–11 m) vs. 8.2 m (95% CI 7.4–9.6 m) (*p* = 0.019)	2002, Rosell et al. [44]
ECOG 0–2	62	Cisplatin based regimen (Cisplatin + Gemcitabine/Vinorelbine) vs. Gemcitabine + Vinorelbine	503	30% vs. 25% (*p* = 0.30)	38 w vs. 32 w (HR = 1.15; 90% CI 0.96–1.37; one-sided, *p* = 0.08)	2003, Gridelli et al. [45]
WHO PS 0–2	61 vs. 62	Cisplatin + Docetaxel vs. Gemcitabine + Docetaxel	441	71 (3 + 68)/71/53; 34.6% (95%CI 28.1–41.1%) vs. 67 (2 + 65)/67/58; 33.3% (95%CI 26.8–39.9%) * No statistically significant difference in both ORRs	10 m (95%CI 0.5–37.5 m) vs. 9.5 m (95% CI 1–36 m) (*p* = 0.980)	2001, Georgoulias et al. [46]
ECOG PS 0–2	63.0 vs. 63.6	Pemetrexed + Cisplatin vs. Docetaxel + Cisplatin	156	24;35.2% vs. 24;37.5% * No statistically significant difference	11.7 m (95% CI, 8.6–14.8 m) vs. 13.3 m (95% CI, 8.1–18.5 m) (*p* > 0.5)	2017, Park et al. [47]
ECOG 0–2	56.8 vs. 57.5	Nedaplatin + Gemcitabine vs. Carboplatin + Gemcitabine	49	9 (0 + 9)/13/2; 37.5% vs. 6 (0 + 6)/15/4; 24% (*p* = 0.305)	17.5 m (95% CI 10.8–24.2 m) vs. 17 m (95% CI 12.1–21.9 m) (*p* = 0.961)	2012, Yang et al. [48]

* “Complete Response” (CR): Disappearance of all target lesions; “Partial Response” (PR): 30% or more decrease in the sum of diameter of target lesions compared to baseline diameter; “Stable Disease” (SD): Neither PR nor PD; “Progressive Disease” (PD): 20% or more increase in sum of diameter of target lesions compared to baseline diameter; “Objective response” = CR + PR [31]. ** “Performance Score” (PS): Eastern Cooperative Oncology Group (ECOG) PS, World Health Organization (WHO) PS, Karnofsky PS (KPS). *** “Overall Survival” (OS), “Median Survival Time” (MST).

**Table 3:** Doublet chemotherapy vs triplet chemotherapy.

PS **	Median Age	Regimen	Patients (n)	Objective Response (CR+PR)/SD/PD; ORR (%) *	Median OS/MST ***	Study
ECOG 0–2	63	Gemcitabine + Cisplatin (GP) vs. Gemcitabine + Vinorelbine (GN) vs. Gemcitabine + Ifosfamide + Cisplatin (GIP) vs. Gemcitabine + Ifosfemide + Vinorelbine (GIN)	433	Platinum based vs. Non-platinum based: 66 (4 + 62)/77/29; 31% (95% CI 25–37%) vs. 52 (4 + 48)/71/39; 24% (95% CI 19–30%) (OR = 0.72, 95% CI 0.47–1.10, *p* = 0.124) Doublet vs. Triplet therapy: 61 (4 + 57)/69/34;29% (95% CI 23–35%) vs. 57 (4 + 57)/79/34;28% (95% CI 21–33%) (OR = 0.86, 95% CI 0.56–1.32, *p* = 0.487)	Platinum based vs. Non-platinum based: 11.3 m (95% CI 9.8–12.7 m) vs. 9.7 m (95% CI 8.7–10.8 m) (HR = 1.23, 95% CI 1.01–1.49, *p* = 0.044) Doublet vs. Triplet therapy: 10.4 m (95% CI 9.4–12.2 m) vs. 10.3 m (95% CI 9.2–11.8 m) (HR = 1.03, 95% CI 0.85–1.25, *p* = 0.781)	2012, Boni et al. [50]
WHO PS 0–2	62	Paclitaxel + Carboplatin vs. Gemcitabine + Paclitaxel + Carboplatin	324	30 (0 + 30)/48/72; 20% vs. 69 (10 + 59)/43/46; 43.6% (*p* ≤ 0.0001)	8.3 m vs. 10.8 m (*p* = 0.044, HR = 1.31, 95% CI 1.02–1.68)	2006, Paccagnella et al. [51]
ECOG PS 0–1	62	Cisplatin + Gemcitabine + Vinorelbine (PGV) vs. Cisplatin + Gemcitabine (PG) vs. Cisplatin + Vinorelbine (PV)	180	28 (2 + 26)/17/15; 47% (95% CI 34–60%) vs. 15 (0 + 15)/15/30; 25% (95% CI 15–38%) vs. 18 (0 + 18)/22/20; 30% (95% CI 19–43%)	51 w vs. 42 w vs. 35 w PGV vs. PV: HR = 0.35 (95% CI 0.16 to 0.77, *p* < 0.0058)	2000, Pasquale et al. [52]
ECOG PS 0–1	NR	Paclitaxel + Carboplatin + Bevacizumab vs. Paclitaxel + Carboplatin	878	59; 15% vs. 133; 35% (*p* < 0.001)	12.3 m vs. 10.3 m (HR = 0.79, 95% CI 0.67–0.92, *p* = 0.003)	2006, Sandler et al. [53]
ECOG 0–2	59 vs. 59 vs. 60	Cisplatin + Gemcitabine (CG) vs. Cisplatin + Gemcitabine + Vinorelbine (CGV) vs. Sequential doublet of Gemcitabine + Vinorelbine followed by Vinorelbine + Ifosfamide (GV-VI)	570	77 (4 + 73)/40/38; 42% vs. 77 (3 + 74)/35/38; 41% vs. 50 (2 + 48)/62/43; 27% (CG vs. CGV, *p* = 0.4) (CG vs. GV-VI, *p* = 0.003) CGV vs. GV-VI, *p* = 0.001)	9.3 m (95% CI 8.1–10.5 m) vs. 8.2 m (95% CI 7–9.4 m) vs. 8.1 m (95% CI 6.9–9.2 m) (Not statistically significant)	2003, Alberola et al. [54]
ECOG 0–1	59 vs. 57 vs. 59	Placebo + Cisplatin + Gemcitabine (CG) vs. Bevacizumab 7.5 mg/kg + Cisplatin + Gemcitabine (CGB7.5) vs. Bevacizumab 15 mg/kg + Cisplatin + Gemcitabine (CGB15)	1043	20.1% vs. 34.1% vs. 30.4% (CG vs. CGB7.5, *p* < 0.0001) (CG vs. CGB15, *p* = 0.0023)	With post-study therapy: 13.1 m vs. 13.6 m vs. 13.4 m (CG vs. CGB7.5 = HR 0.93, 95% CI 0.78–1.11, *p* = 0.420) (CG vs. CGB15 = HR 1.03, 95% CI 0.86–1.23, *p* = 0.76) Without poststudy therapy: CG vs. CGB (7.5 + 15) 7.3 m vs. 8.7 m (HR 0.84, *p* = 0.20)	2009, Reck et al. [55]
						2010, Reck et al. [56]
ECOG 0–1	57 vs. 56	Carboplatin + Paclitaxel + Bevacizumab vs. Carboplatin + Paclitaxel + Placebo	276	74 (0 + 74)/55/2; 54% (95% CI 46–63%) vs. 35 (0 + 35)/83/10; 26% (95% CI 19–35%) (*p* < 0.001)	24.3 m vs. 17.7 m (HR 0.68; 95% CI 0.50–0.93, *p* = 0.0154)	2015, Zhou et al. [49]
ECOG 0–2	58.8	Bevacizumab + standard-of-care first-line chemotherapy: Carboplatin doublet vs. Cisplatin doublet vs. Non-platinum doublets vs. Monotherapy vs. Triplet and quadruplet chemotherapy regimen	2212	Post-baseline disease assessment (2036 patients): 1049 (65 + 984)/756/NR; 51%	Overall population: Median OS 14.6 m (95% CI 13.8–15.3 m 14.3 m (95% CI 13.2–15.6 m) vs. 14.7 m (95% CI 13.7–16.0 m) vs. 8.1 m (95% CI 5.7–13.0 m) vs. 9.4 m (95% CI 5.3–14.7 m) vs. 13.8 m (95% CI 4.4–21.7 m)	2010, Crinò, et al. [57]
ECOG PS 0–1	64.6 vs. 64.9	Pemetrexed + Carboplatin + Bevacizumab vs. Paclitaxel + Carboplatin + Bevacizumab	939	34.1% vs. 33.0%	12.6 m vs. 13.4 m (HR = 1.00, 95% CI 0.86–1.16, *p* = 0.949)	2013, Patel et al. [58]

* “Complete Response” (CR): Disappearance of all target lesions; “Partial Response” (PR): 30% or more decrease in the sum of diameter of target lesions compared to baseline diameter; “Stable Disease” (SD): Neither PR nor PD; “Progressive Disease” (PD): 20% or more increase in sum of diameter of target lesions compared to baseline diameter; “Objective response” = CR + PR [31]. ** “Performance Score” (PS): Eastern Cooperative Oncology Group (ECOG) PS, World Health Organization (WHO) PS, Karnofsky PS (KPS). *** “Overall Survival” (OS), “Median Survival Time” (MST).

**Table 4:** Immune checkpoint inhibitor in first-line treatment.

PS **	Median Age	Regimen	Patients (n)	Objective Response (CR + PR)/SD/PD; ORR (%) *	Median OS/MST ***	Study
ECOG 0–1	63	Pembrolizumab vs. Carboplatin + Paclitaxel/Pemetrexed	1275	NR	TPS 50% or greater: 20 m (95% CI 15.4–24.9 m) vs. 12.2 m (95% CI 10.4–14.2 m) (HR 0.69, 95% CI 0.56–0.85, *p* = 0.003) TPS 20% or greater: 17.7 m (95% CI 15.3–22.1 m) vs. 13.0 m (95% CI 11.6–15.3 m) (HR 0.77, 95% CI 0.64–0.92, *p* = 0.002) TPS 1% or greater: 16.7 m (95% CI 13.9–19.7 m) vs. 12.1 m (95% CI 11.3–13.3 m) (HR 0.81, 95% CI 0.73–0.93, *p* = 0.0018	2019, Mok et al. [59]
ECOG 0–1	64.5 vs. 66.0	Pembrolizumab vs. Platinum-based regimen (Carboplastin + Pemetrexed, Cisplatin + Pemetrexed, Carboplastin + Gemcitabine, Cisplatin + Gemcitabine, Carbolastin + Paclitaxel)	305	44.8% (95% CI 36.8–53.0%) vs. 27.8% (95% CI 20.8–35.7%)	Median OS not met. HR 0.60, 95% CI 0.41–0.89, *p* = 0.005	2016, Reck et al. [60]
				NR	30 m (95% CI 18.3–NR) vs. 14.2 m (95% CI 9.8–19.0 m) (HR 0.63, 95% CI 0.47–0.86, *p* = 0.002)	2019, Reck et al. [61]
ECOG 0–1	65 vs. 63.5	Cisplatin/Carboplatin + Pemetrexed + Pembrolizumab vs. Cisplatin/Carboplatin + Pemetrexed + Placebo	616	47.6% (95% CI 42.6–52.5%) vs. 18.9% (95% CI 13.8–25.0%) (*p* < 0.001)	Immature result vs. 11.3 m (95% CI 8.7–15.1%) (HR 0.49, 95% CI 0.38–0.64, *p* ≤ 0.001)	2018, Gandhi et al. [62]
				197 (4 + 193)/150/37; 48% (95% CI 43.1–53.0%) vs. 40 (1 + 39)/105/36; 19.4% (95% CI 14.2–25.5%)	Median study follow-up = 23.1 m 22 m (95% CI 19.5–25.2 m) vs. 10.7 m (95% CI 8.7–13.6 m) (HR 0.56, 95% CI 0.45–0.70	2020, Gadgeel et al. [63]
ECOG 0–1	63 vs. 65	Nivolumab vs. Platinum doublet chemotherapy (Pemetrexed + Carboplatin, Pemetrexed + Cisplatin, Gemcitabine + Carboplatin, Gemcitabine + Cisplatin, Paclitaxel + Carboplatin)	541	55 (4 + 51)/81/58; 26% (95% CI 20–33%) vs. 71 (1 + 70)/100/21; 33% (95% CI 27–40%) (OR 0.70, 95% CI 0.46–1.06)	14.4 m (95% CI 11.7–17.4 m) vs. 13.2 m (95% CI 10.7–17.1 m) (HR 1.02, 95% CI 0.80–1.30	2017, Carbone et al. [64]
ECOG 0–1	66 vs. 65 vs. 65	Atezolizumab + Carboplatin + Paclitaxel (ACP) vs. Atezolizumab + Carboplatin + Nab-paclitaxel (ACnP) vs. Carboplatin + nab-paclitaxel (CnP)	1021	ACnP vs. CnP: 170 (8 + 162)/107/25; 49.7% (95% CI 44.3–55.1%) vs. 139 (5 + 134)/120/48; 41.0% (95% CI 35.7–46.5%)	ACnP vs. CnP: 14.2 m (95% CI 12.3–16.8 m) vs. 13.5 m (95% CI 12.2–15.1 m) (HR 0.88, 95% CI 0.73–1.05, *p* = 0.1581)	2020, Jotte et al. [65]
ECOG 0–1	64 vs. 65	Atezolizumab vs. Cisplatin/Carboplatin + Pemetrexed/Gemcitabine	572	High PD-L1 expression: 38.3% (95% CI 29.1–48.2%) vs. 28.6% (95% CI 19.9–38.6%) High or intermediate PF-L1 expression: 30.7% (95% CI 23.8–38.3%) vs. 32.1% (95% CI 25.0–39.9%) Any PD-L1 expression: 29.2% (95% Ci 24.0–35.0%) vs. 31.8% (95% CI 26.3–37.6%)	High PD-L1 expression: 20.2 m vs. 13.1 m (HR 0.59, 95% CI 0.40–0.89, *p* = 0.01) High or intermediate PF-L1 expression: 18.2 m vs. 14.9 m (HR 0.72, 95% CI 0.52–0.99, *p* = 0.044) Any PD-L1 expression: 17.5 m vs. 14.1 m (HR 0.83, 95% CI 0.65–1.07)	2020, Giaccone et al. [66]
ECOG 0–1	65	Pembrolizumab + Carboplatin + Paclitaxel/Nab-paclitaxel vs. Placebo + Carboplatin + Paclitaxel/Nab-paclitaxel	559	161; 57.9% (95% CI 51.9–63.8%) vs. 108; 38.4% (95% CI 32.7–44.4%)	15.9 m (95% CI 13.2-NR) vs. 11.3 m (95% CI 9.5–14.8 m) (HR 0.64, 95% CI 0.49–0.85, *p* < 0.001)	2018, Paz-Ares et al. [67]
ECOG 0–1	63 vs. 63	Atezolizumab + Bevacizumab + Carboplatin + Paclitaxel (ABCP) vs. Bevacizumab + Carboplatin + Paclitaxel (BCP)	1202	224 (13 + 211)/77/18; 63.5% (95% CI 58.2–68.5%) vs. 159 (4 + 155)/115/27; 48.0% (95% CI 42.5–53.6%)	19.2 m vs. 14.7 m (HR 0.78, 95% CI 0.64–0.96, *p* = 0.02)	2018, Socinski et al. [68]

* “Complete Response” (CR): Disappearance of all target lesions; “Partial Response” (PR): 30% or more decrease in the sum of diameter of target lesions compared to baseline diameter; “Stable Disease” (SD): Neither PR nor PD; “Progressive Disease” (PD): 20% or more increase in sum of diameter of target lesions compared to baseline diameter; “Objective response” = CR + PR [31]. ** “Performance Score” (PS): Eastern Cooperative Oncology Group (ECOG) PS, World Health Organization (WHO) PS, Karnofsky PS (KPS). *** “Overall Survival” (OS), “Median Survival Time” (MST).

**Table 5:** Treatment of tumors with positive EGFR mutation.

PS **	Median Age	Regimen	Patients (n)	Objective Response (CR + PR)/SD/PD; ORR (%) *	Median OS/MST ***	Study
ECOG 0–1	63	Erlotinib + Chemotherapy (Carboplatin + Paclitaxel) vs. Placebo + Chemotherapy (Carboplatin + Paclitaxel)	1079	21.5% vs. 19.3% (*p* = 0.36)	10.6 m vs. 10.5 m (HR 0.995, 95% CI 0.86–1.16, *p* = 0.95)	2005, Herbst et al. [69]
WHO 0–2	62 vs. 61 vs. 63	Chemotherapy (Paclitaxel + Carboplatin) + 500 mg/d Gefitinib vs. Chemotherapy + 250 mg/d Gefitinib vs. Chemotherapy + Placebo	1037	CR rate; ORR: 0.6%; 30% vs. 2.6%; 30.4% vs. 1.2%; 28.7% (No statistically significant difference)	8.7 m vs. 9.8 m vs. 9.9 m (*p* = 0.6385)	2004, Herbst et al. [70]
ECOG 0–2	NR	Erlotinib vs. Chemotherapy (Cisplatin/Carboplatin + Docetaxel/Gemcitabine)	173	49 (2 + 47); 64% vs. 13 (0 + 13); 18% (OR 7.5, 95% CI 3.6–15.6, *p* < 0.0001)	19.3 m (95% CI 14.7–26.8 m) vs. 19.5 m (95% CI 16.1 m–NR) (HR 1.04, 95% CI 0.65–1.68, *p* = 0.87)	2012, Rosell et al. [71]
ECOG 0–2	63.9 vs. 62.6	Gefitinib vs. Carboplatin + Paclitaxel	230	73.7% vs. 30.7% (*p* < 0.001)	30.5 m vs. 23.6 m (*p* = 0.31)	2010, Maemondo et al. [72]
ECOG 0–1	58	Afatinib vs. Gemcitabine + Cisplatin	364	162; 66.9% vs. 28; 23% (OR 7.28, 95% CI 4.36–12.18, *p* < 0.0001)	NR	2014, Wu et al. [73]
					23.1 m (95% CI 20.4–27.3 m) vs. 23.5 m (95% CI 18.0–25.6 m) (HR 0.93, 95% CI 0.72–1.22, *p* = 0.61	2015, Yang et al. [74]
ECOG 0–1	61.5 vs. 61.0	Afatinib vs. Cisplatin + Pemetrexed	345	56% vs. 23% (*p* = 0.001)	NR	2013, Sequist et al. [75]
					28.2 m (95% CI 24.6–33.6 m) vs. 28.2 m (95% CI 20.7–33.2 m) (HR 0.88, 95% CI 0.66–1.17, *p* = 0.39)	2015, Yang et al. [74]
WHO PS 0–1	NR	Osimertinib vs. Gefitinib/Erlotinib	556	80% (95% CI 75–85%) vs. 76% (95% CI 70–81%)	NR	2017, Ramalingam et al. [76]
					38.6 m (95% CI 34.5–41.8 m) vs. 31.8 m (95% CI 26.6–36.0 m) (HR 0.799, 95% CI 0.641–0.997, *p* = 0.0462)	2019, Ramalingam et al. [77]
WHO PS 0–1	64	Gefitinib vs. Cisplatin + Docetaxel	106	36; 62.1% vs. 19; 32.2% (*p* < 0.0001)	NR	2010, Mitsudomi et al. [78]
					34.9 m (95% CI 26.1–39.5 m) vs. 37.3 m (95% CI 31.2–45.5 m) (HR 1.252, 95% CI 0.883–1.775, *p* < 0.2070)	2014, Yoshioka et al. [79]

* “Complete Response” (CR): Disappearance of all target lesions; “Partial Response” (PR): 30% or more decrease in the sum of diameter of target lesions compared to baseline diameter; “Stable Disease” (SD): Neither PR nor PD; “Progressive Disease” (PD): 20% or more increase in sum of diameter of target lesions compared to baseline diameter; “Objective response” = CR + PR [31]. ** “Performance Score” (PS): Eastern Cooperative Oncology Group (ECOG) PS, World Health Organization (WHO) PS, Karnofsky PS (KPS). *** “Overall Survival” (OS), “Median Survival Time” (MST).

**Table 6:** Treatment of tumors with positive ALK translocation.

PS **	Median Age	Regimen	Patients (n)	Objective Response (CR + PR)/SD/PD; ORR (%) *	Median OS/MST ***	Study
ECOG PS 0–2	52 vs. 54	Crizotinib vs. Chemotherapy (Pemetrexed + Cisplatin/Carboplatin)	343	128 (3 + 125)/29/8; 74% (95% CI 67–81%) vs. 77 (2 + 75)/63/21; 45% (95% CI 37–53%) (*p* < 0.001)	HR 0.82 (95% CI 0.54–1.26, *p* = 0.36) (Immature results)	2014, Solomon et al. [81]
					NR (95% CI 45.8 m–NR) vs. 47.5 m (95% CI 32.2–NR) (HR 0.760, 95% CI 0.548–1.053, *p* = 0.0978)	2018, Solomon et al. [82]
ECOG PS 0–2	48 vs. 50	Crizotinib vs. Pemetrexed + Cisplatin/Carboplatin	207	91 (3 + 88); 87.5% (95% CI 79.6–93.2%) vs. 47 (0 + 47); 45.6% (95% CI 35.8–55.7%) (*p* < 0.001)	28.5 m (95% CI 26.4 m–NR) vs. 27.7 m (95% CI 23.9 m–NR) (HR 0.897, 95% CI 0.556–1.445, *p* = 0.327)	2018, Wu et al. [80]
ECOG PS 0–2	53.8 vs. 56.3	Crizotinib vs. Alectinib	303	114 (2 + 112)/24/NR; 75.5% (95% CI 67.8–82.1%) vs. 126 (6 + 120)/9/NR; 82.9% (95% CI 76.0–88.5%) (*p* = 0.09)	HR = 0.76, 95% CI 0.48–1.20, *p* = 0.24	2017, Peters et al. [83]
ECOG PS 0–2	59.1 vs. 55.6	Lorlatinib vs. Crizotinib	296	113 (4 + 109)/19/10; 76% (95% CI 68–83%) vs. 85 (0 + 85)/41/7; 58% (95% CI 49–66%) (OR 2.25, 95% CI 1.35–3.89)	HR = 0.72, 95% CI 0.41–1.25	2020, Shaw et al. [84]

* “Complete Response” (CR): Disappearance of all target lesions; “Partial Response” (PR): 30% or more decrease in the sum of diameter of target lesions compared to baseline diameter; “Stable Disease” (SD): Neither PR nor PD; “Progressive Disease” (PD): 20% or more increase in sum of diameter of target lesions compared to baseline diameter; “Objective response” = CR + PR [31]. ** “Performance Score” (PS): Eastern Cooperative Oncology Group (ECOG) PS, World Health Organization (WHO) PS, Karnofsky PS (KPS). *** “Overall Survival” (OS), “Median Survival Time” (MST).

**Table 7:** Treatment of tumors with positive ROS1, BRAF and HER2 mutations.

PS **	Median Age	Regimen	Patients (n)	Objective Response (CR+PR)/SD/PD; ORR (%) *	Median OS/MST ***	Study
ECOG 0–2	62	Crizotinib	93	*ROS1* translocation cohort: 17; 47.2%	*ROS* translocation cohort: 17.2 m (95% CI 6.8–32.8 m)	2019, Moro-Sibilot et al. [86]
ECOG 0–1	NR	Crizotinib	53	38 (6 + 32)/10/3; 72% (95% CI 58–83%)	51.4 m (95% CI 29.3 m–NR)	2019, Shaw et al. [85]
ECOG 0–2	66	Dabrafenib	84	≥second-line patients: 21(NR)/13/23; 33% (95% CI 23–45%)	12.7 m (95% CI 7.3–16.9 m)	2016, Planchard et al. [87]
ECOG 0–2	BRAF^V600^ = 68 BRAF^Non-V600^ = 65	Vemurafenib	118	BRAF^V600^: 43; 44.8% *BRAF^Non-V600^*: No tumor response observed	*BRAF^V600^*: 10 m (95% CI 6.8–15.7 m) *BRAF^Non-V600^*: 5.2 m (95% CI 2.8–18.7 m)	2020, Mazieres et al. [88]
ECOG 0–2	64	Debrafenib + Trametinib	59	36 (0 + 36)/4/8; 63.2% (95% CI 49.3–75.6%)	NR	2016, Planchard et al. [89]
					18.2 m (95% CI 14.3 m–NR)	2017, Planchard et al. [90]
NR	57	Pyrotinib	60	31.7%	NR	2019, Gao et al. [91]
NR	42	Trastuzumab deruxtecan	42	41.9%	NR	2020, Smit et al. [92]
NR	60	Poziotinib	90	27.8%	NR	2020, Socinski et al. [93]

* “Complete Response” (CR): Disappearance of all target lesions; “Partial Response” (PR): 30% or more decrease in the sum of diameter of target lesions compared to baseline diameter; “Stable Disease” (SD): Neither PR nor PD; “Progressive Disease” (PD): 20% or more increase in sum of diameter of target lesions compared to baseline diameter; “Objective response” = CR + PR [31]. ** “Performance Score” (PS): Eastern Cooperative Oncology Group (ECOG) PS, World Health Organization (WHO) PS, Karnofsky PS (KPS). *** “Overall Survival” (OS), “Median Survival Time” (MST).

**Table 8:** ECOG Performance Status Score [94,95].

Score	Definition
0	Fully active without any restrictions to daily activities.
1	Able to ambulate and carry out light works only.
2	Able to ambulate >50% of waking hours and perform self-care.
3	Confined to bed/chair >50% of waking hours and limited self-care.
4	Total confinement to bed/chair and unable to perform any self-care.

**Table 9:** Common therapeutics used in treating advanced NSCLC and their common dosages.

Drug	Dosage as Monotherapy	Dosage in Combination Therapy
Cisplatin	100 mg/m^2^	50–120 mg/m^2^
Carboplatin *	NR	AUC 5 or 6 mg/(mL × min)
Nedaplatin	NR	80–100 mg/m^2^
Gemcitabine	NR	1000–1250 mg/m^2^
Docetaxel	75 mg/m^2^	60–100 mg/m^2^
Paclitaxel	NR	135–225 mg/m^2^
Vinorelbine	30 mg/m^2^	25 or 30 mg/m^2^
Pemetrexed	500 mg/m^2^	500 mg/m^2^
Bevacizumab	NR	15 or 7.5 mg/kg
Pembrolizumab	200 mg	200 mg
Atezolizumab	1200 mg	1200 mg
Gefitinib	250 mg	250 or 500 mg
Erlotinib	150 mg	150 mg
Afatinib	40 mg	NR
Osimertinib	80 mg	NR
Crizotinib	250 mg	NR

* Dosage of carboplatin can be converted to milligram using the Calvert formula: Total Dose (mg) = (target AUC) × (GFR + 25) [98].

**Table 10:** Median cycles of treatment administered/Median duration of treatment.

Types of Regimen	Median Cycles of Treatment Administered/Median Duration of Treatment
Platinum-based doublet ^1^	4 cycles
Platinum-based triplet ^2^	5–7 cycles
*PD-1*/*PD-L1* inhibitors ^3^	8–10 cycles
*EGFR TK* inhibitors ^4^	3–13 m
Crizotinib	10.7–15.6 m

^1^ “Platinum-based doublet”: Cisplatin/carboplatin/nedaplatin + gemcitabine/docetaxel/paclitaxel/vinorelbine; ^2^ “Platinum-based triplet”: Platinum-based doublet + bevacizumab; ^3^ “*PD-1/PD-L1* inhibitors”: Pembrolizumab or atezolizumab; ^4^ “*EGFR TK* inhibitor”: Gefitinib/erlotinib/afatinib/osimertinib.

## Data Availability

Not applicable.

## Supplementary Materials

The following are available online at https://www.mdpi.com/article/10.3390/medicina57111252/s1, Table S1: Studies with top 10 highest “Complete Response” rate, Table S2: Studies with top 10 highest overall survival.
