# Supplementary material for: Clinical Outcomes of Chemotherapeutic Molecules as Single and Multiple Agents in Advanced Non-Small-Cell Lung Carcinoma (NSCLC) Patients
Source: Medicina (Kaunas). 2021 Nov 16;57(11):1252. doi: 10.3390/medicina57111252 (PMC8618045; doi:10.3390/medicina57111252)
Supplement: Supplementary file 1 [file medicina-57-01252-s001.zip › medicina-1401471-supplementary.pdf]

Supplementary Table S1. Studies with top 10 highest "Complete Response" rate.

PS	Median Age	Regimen	Median Cycle of Treatment, (n)/Median Duration of Treatment	Patients (n)	ORR; CRR * (%)	Study
0–1	63	Cisplatin monotherapy 100 mg/m^2 vs. Cisplatin 100 mg/m^2 + Vinorelbine 25 mg/m^2	2 vs. 3	415	12%; 0% vs. 26%; 2% (P.0002)	1998, Wozniak et al. [1]
KPS 70–100	61 vs. 59 vs. 61	Docetaxel 75 mg/m^2 + Cisplatin 75 mg/m^2 (DC) vs. Docetaxel 75 mg/m^2 + Carboplatin AUC 6 (DCb) vs. Vinorelbine 25 mg/m^2 + Cisplatin 100 mg/m^2 (VC)	5 vs. 6 vs. 4	1218	31.6% (95% CI 27.1–36.4%); 2% vs. 23.9% (95% CI 19.8–28.3%); 1.2% vs. 24.5% (95% CI 20.4–29.0%); 2%<br><br>DC vs. VC ($p = 0.029$) DCb vs. VC ($p = 0.870$)	2003, Fossella et al. [2]
NR	56.28 vs. 55.01	Nedaplatin Group (Nedaplatin 75 mg/m^2 + Pemetrexed/ Docetaxel/Gemcitabine/Vinorelbine/Paclitaxel) vs. Cisplatin Group (Cisplatin 75 mg/m^2 + Pemetrexed/Docetaxel/Gemcitabine/Vinorelbine/Paclitaxel)	4.0 vs. 3.0	619	48.6%; 4.1% vs. 35.1%; 3.1% ($p < 0.01$)	2014, Li et al. [3]
ECOG 0–2	59 vs. 59 vs. 60	Cisplatin 100 mg/m^2 + Gemcitabine 1250 mg/m^2 (CG) vs. Cisplatin 100 mg/m^2 + Gemcitabine 1000 mg/m^2 + Vinorelbine 25 mg/m^2 (CGV) vs. Sequential doublet of Gemcitabine 1000 mg/m^2 + Vinorelbine 30 mg/m^2 followed by Vinorelbine 30 mg/m^2 + Ifosfamide 3 g/m^2 (GV-VI)	4	570	42%; 2% vs. 41%; 2% vs. 27%; 1%<br><br>(CG vs. CGV, $p = 0.4$) (CG vs. GV-VI, $p = 0.003$) CGV vs. GV-VI, $p = 0.001$)	2003, Alberola et al. [4]

WHO PS 0–2	62	Paclitaxel 200 mg/m^2 + Carboplatin AUC 6 vs. Gemcitabine 1000 mg/m^2 + Paclitaxel 200 mg/m^2 + Carboplatin AUC 6	4 vs. 5	324	20%; 0% vs. 43.6%; 6.3% ($p \leq 0.0001$)	2006, Paccagnella et al. [5]
ECOG 0–2	NR	Erlotinib 150 mg vs. Chemotherapy (Cisplatin 75 mg/m^2/carboplatin AUC 6 + Docetaxel 75 mg/m^2/Gemcitabine 1000 mg/m^2)	8.2 m vs. 2.8 m/4 cycles	173	64%; 3.0% vs. 18%; 0%<br>(OR 7.5, 95% CI 3.6–15.6, $p < 0.0001$)	2012, Rosell et al. [6]
ECOG 0–2	63.9 vs. 62.6	Gefitinib 250 mg vs. Carboplatin AUC 6 + Paclitaxel 200 mg/m^2	308 days vs. 4 cycles	230	73.7%; 4.4% vs. 30.7%; 0%<br>($p < 0.001$)	2010, Maemondo et al. [7]
ECOG 0–1	58	Afatinib 40 mg vs. Gemcitabine 1000 mg/m^2 + Cisplatin 75 mg/m^2	398 days vs. 89 days/4 cycles	364	66.9%; 1.2% vs. 23%; 0%<br>(OR 7.28, 95% CI 4.36–12.18, $p < 0.0001$)	2014, Wu et al. [8]<br>2015, Yang et al. [9]
ECOG PS 0–2	52 vs. 54	Crizotinib 250 mg vs. Chemotherapy (Pemetrexed 500 mg/m^2 + Cisplatin 75 mg/m^2/Carboplatin AUC 5–6)	10.9 m vs. 4.1 m/6 cycles	343	74% (95% CI 67–81%); 2% vs. 45% (95% CI 37–53%); 1%<br>($p < 0.001$)	2014, Solomon et al. [10]<br>2018, Solomon et al. [11]
ECOG PS 0–2	48 vs. 50	Crizotinib 250 mg vs. Pemetrexed 500 mg/m^2 + Cisplatin 75 mg/m^2/Carboplatin 5–6 AUC	15.6 m vs. 4.2 m	207	87.5% (95% CI 79.6–93.2%); 2.9% vs. 45.6% (95% CI 35.8–55.7%); 0%<br>($p < 0.001$)	2018, Wu et al. [12]

* CRR: Complete Response Rate; ORR: Objective Response Rate.

Supplementary Table S2. Studies with top 10 highest overall survival.

PS	Median Age	Regimen	Median Cycle of Treatment (n)	Patients (n)	Median OS/MST *	Study
NR	56.28 vs. 55.01	Nedaplatin Group (Nedaplatin 75 mg/m^2 + Pemetrexed/Docetaxel/ Gemcitabine/Vinorelbine/Paclitaxel) vs. Cisplatin Group (Cisplatin 75 mg/m^2 + Pemetrexed/Docetaxel/ Gemcitabine/Vinorelbine/Paclitaxel)	4.0 vs. 3.0	619	(14.783 ± 1.092) m vs. (13.502 ± 2.327) m ($p < 0.01$)	2014, Li et al. [3]
NR	NR	Nedaplatin 80 mg/m^2 + Gemcitabine 1000 mg/m^2/Paclitaxel 135–	4.1 vs. 3.3	392	20 m (95% CI 17.0–23.0 m)	2015, Shan et al. [13]

		175 mg/m^2/Navelbine 25 mg/m^2/Docetaxel 75 mg/m^2/Cyclophosphamide 600 mg/m^2/Doxorubicin 50 mg/m^2 vs. Cisplatin 80 mg/m^2 + Gemcitabine 1000 mg/m^2/Paclitaxel 135–175 mg/m^2/Navelbine 25 mg/m^2/Docetaxel 75 mg/m^2/Cyclophosphamide 600 mg/m^2/Doxorubicin 50 mg/m^2			vs. 15 m (95% CI 13.4–16.6 m)<br><br>($p = 0.022$)	
ECOG PS 0–1	NR	Paclitaxel 200 mg/m^2 + Carboplatin AUC 6 + Bevacizumab 15 mg/kg vs. Paclitaxel 200 mg/m^2 + Carboplatin AUC 6	7 vs. 5	878	12.3 m vs. 10.3 m<br><br>(HR = 0.79, 95% CI 0.67–0.92, $p = 0.003$)	2006, Sandler et al. [14]
ECOG PS 0–1	62	Cisplatin 50 mg/m^2 + Gemcitabine 1000 mg/m^2 + Vinorelbine 25 mg/m^2(PGV) vs. Cisplatin 100 mg/m^2 + Gemcitabine 1000 mg/m^2 (PG) vs. Cisplatin 120 mg/m^2 + Vinorelbine 30 mg/m^2 (PV)	NR	180	51 w vs. 42 w vs. 35 w<br><br>PGV vs. PV: HR = 0.35 (95% CI 0.16 to 0.77, $p < 0.0058$)	2000, Pasquale et al. [15]
ECOG PS 0–1	57 vs. 56	Carboplatin AUC 6 + Paclitaxel 175 mg/m^2 + Bevacizumab 15 mg/kg vs. Carboplatin AUC 6+ Paclitaxel 175 mg/m^2 + Placebo	11 vs. 8	276	24.3 m vs. 17.7 m<br><br>(HR 0.68; 95% CI 0.50–0.93, $p = 0.0154$)	2015, Zhou et al. [16]
ECOG 0–1	63	Pembrolizumab 200 mg vs. Carboplatin AUC 5–6 mg/mL/min + Paclitaxel 200 mg/m^2/Pemetrexed 500 mg/m^2	9 vs. 6	1275	TPS 50% or greater: 20 m (95% CI 15.4–24.9 m) vs. 12.2 m (95% CI 10.4–14.2 m) (HR 0.69, 95% CI 0.56–0.85, $p = 0.003$)<br><br>TPS 20% or greater: 17.7 m (95% CI 15.3–22.1 m) vs. 13.0 m (95% CI 11.6–15.3 m)	2019, Mok et al. [17]

					(HR 0.77, 95% CI 0.64–0.92, $p = 0.002$)<br><br>TPS 1% or greater: 16.7 m (95% CI 13.9–19.7 m) vs. 12.1 m (95% CI 11.3–13.3 m) (HR 0.81, 95% CI 0.73–0.93, $p = 0.0018$	
ECOG 0–1	64.5 vs. 66.0	Pembrolizumab 200 mg vs. Platinum-based regimen (Carboplatin AUC 5–6 + Pemetrexed 500 mg/m², Cisplatin 75 mg/m² + Pemetrexed 500 mg/m², Carboplatin AUC 5–6 + Gemcitabine 1250 g/m², Cisplatin 75 mg/m² + Gemcitabine 1250 mg/m², Carboplatin AUC 5–6 + Paclitaxel 200 mg/m²)	10.5 vs. 4	305	Median OS not met.<br><br>HR 0.60, 95% CI 0.41–0.89, $p = 0.005$	2016, Reck et al. [18]
					30 m (95% CI 18.3–NR) vs. 14.2 m (95% CI 9.8–19.0 m)<br><br>(HR 0.63, 95% CI 0.47–0.86, $p = 0.002$	2019, Reck et al. [19]
ECOG 0–1	64 vs. 65	Atezolizumab 1200 mg vs. Cisplatin 75 mg/m² / Carboplatin AUC 6 + Pemetrexed 500 mg/m²/Gemcitabine 1250 mg/m²	NR	572	High PD-L1 expression: 20.2 m vs. 13.1 m (HR 0.59, 95% CI 0.40–0.89, $p = 0.01$)<br><br>High or intermediate PF-L1 expression: 18.2 m vs. 14.9 m (HR 0.72, 95% CI 0.52–0.99, $p = 0.044$)<br><br>Any PD-L1 expression: 17.5 m vs. 14.1 m (HR 0.83, 95% CI 0.65–1.07)	2020, Giaccone et al. [20]
ECOG 0–1	65	Pembrolizumab 200 mg + Carboplatin AUC 6 + Paclitaxel 200 mg/m²/Nab-paclitaxel 100 mg/m² vs. Placebo + Carboplatin AUC 6 + Paclitaxel 200	Pembrolizumab (8/8), Carboplatin (4/4), Paclitaxel (4)/Nab-paclitaxel (10) vs. Carboplatin (8/6), Paclitaxel	559	15.9 m (95% CI 13.2–NR) vs. 11.3 m (95% CI 9.5–14.8 m)<br><br>(HR 0.64, 95% CI 0.49–0.85, $p < 0.001$)	2018, Paz-Ares et al. [21]

		mg/m²/Nab-paclitaxel 100 mg/m²	(4)/Nab-paclitaxel (10)			
ECOG 0–1	63 vs. 63	Atezolizumab 1200 mg + Bevacizumab 15 mg/kg + Carboplatin AUC 6 + Paclitaxel 200 mg/m² (ABCP) vs. Bevacizumab 15 mg/kg + Carboplatin AUC 6 + Paclitaxel 200 mg/m² (BCP)	Atezolizumab (12), Bevacizumab (10) vs. Bevacizumab (8)	1202	19.2 m vs. 14.7 m (HR 0.78, 95% CI 0.64–0.96, *p* = 0.02)	2018, Socinski et al. [22]

* "Overall Survival" (OS), "Median Survival Time" (MST).

References:

1. Wozniak, A.J.; Crowley, J.J.; Balcerzak, S.P.; Weiss, G.R.; Spiridonidis, C.H.; Baker, L.H.; Albain, K.S.; Kelly, K.; A Taylor, S.; Gandara, D.R.; et al. Randomized trial comparing cisplatin with cisplatin plus vinorelbine in the treatment of advanced non-small-cell lung cancer: A Southwest Oncology Group study. *J. Clin. Oncol*. **1998**, *16*, 2459–2465, doi:10.1200/jco.1998.16.7.2459.
2. Fossella, F.; Pereira, J.R.; Von Pawel, J.; Pluzanska, A.; Gorbounova, V.; Kaukel, E.; Mattson, K.V.; Ramlau, R.; Szczęsna, A.; Fidias, P.; et al. Randomized, Multinational, Phase III Study of Docetaxel Plus Platinum Combinations Versus Vinorelbine Plus Cisplatin for Advanced Non–Small-Cell Lung Cancer: The TAX 326 Study Group. *J. Clin. Oncol*. **2003**, *21*, 3016–3024, doi:10.1200/jco.2003.12.046.
3. Li, C.-H.; Liu, M.-Y.; Liu, W.; Li, D.-D.; Cai, L. Randomized control study of nedaplatin or cisplatin concomitant with other chemotherapy in the treatment of advanced non-small cell lung cancer. *Asian Pac. J. Cancer Prev.* **2014**, *15*, 731–736, doi:10.7314/apjcp.2014.15.2.731.
4. Alberola, V.; Camps, C.; Provencio, M.; Isla, D.; Rosell, R.; Vadell, C.; Bover, I.; Ruiz-Casado, A.; Azagra, P.; Jiménez, U.; et al. Cisplatin Plus Gemcitabine Versus a Cisplatin-Based Triplet Versus Nonplatinum Sequential Doublets in Advanced Non–Small-Cell Lung Cancer: A Spanish Lung Cancer Group Phase III Randomized Trial. *J. Clin. Oncol.* **2003**, *21*, 3207–3213, doi:10.1200/jco.2003.12.038.
5. Paccagnella, A.; Oniga, F.; Bearz, A.; Favaretto, A.; Clerici, M.; Barbieri, F.; Riccardi, A.; Chella, A.; Tirelli, U.; Ceresoli, G.; et al. Adding Gemcitabine to Paclitaxel/Carboplatin Combination Increases Survival in Advanced Non–Small-Cell Lung Cancer: Results of a Phase II-III Study. *J. Clin. Oncol.* **2006**, *24*, 681–687, doi:10.1200/jco.2005.03.2722.
6. Rosell, R.; Carcereny, E.; Gervais, R.; Vergnenegre, A.; Massuti, B.; Felip, E.; Palmero, R.; Garcia-Gomez, R.; Pallares, C.; Sanchez, J.M.; et al. Erlotinib versus standard chemotherapy as first-line treatment for European patients with advanced EGFR mutation-positive non-small-cell lung cancer (EURTAC): A multicentre, open-label, randomised phase 3 trial. *Lancet Oncol.* **2012**, *13*, 239–246, doi:10.1016/s1470-2045(11)70393-x.
7. Maemondo, M.; Inoue, A.; Kobayashi, K.; Sugawara, S.; Oizumi, S.; Isobe, H.; Gemma, A.; Harada, M.; Yoshizawa, H.; Kinoshita, I.; et al. Gefitinib or Chemotherapy for Non–Small-Cell Lung Cancer with Mutated EGFR. *N. Engl. J. Med.* **2010**, *362*, 2380–2388, doi:10.1056/nejmoa0909530.
8. Wu, Y.-L.; Zhou, C.; Hu, C.-P.; Feng, J.; Lu, S.; Huang, Y.; Li, W.; Hou, M.; Shi, J.H.; Lee, K.Y.; et al. Afatinib versus cisplatin plus gemcitabine for first-line treatment of Asian patients with advanced non-small-cell lung cancer harbouring EGFR mutations (LUX-Lung 6): An open-label, randomised phase 3 trial. *Lancet Oncol.* **2014**, *15*, 213–222, doi:10.1016/s1470-2045(13)70604-1.
9. Yang, J.C.-H.; Wu, Y.; Schuler, M.; Sebastian, M.; Popat, S.; Yamamoto, N.; Zhou, C.; Hu, C.-P.; O'Byrne, K.; Feng, J.; et al. Afatinib versus cisplatin-based chemotherapy for EGFR mutation-positive lung adenocarcinoma (LUX-Lung 3 and LUX-Lung 6): Analysis of overall survival data from two randomised, phase 3 trials. *Lancet Oncol.* **2015**, *16*, 141–151, doi:10.1016/s1470-2045(14)71173-8.
10. Solomon, B.J.; Mok, T.; Kim, D.-W.; Wu, Y.-L.; Nakagawa, K.; Mekhail, T.; Felip, E.; Cappuzzo, F.; Paolini, J.; Usari, T.; et al. First-Line Crizotinib versus Chemotherapy in ALK-Positive Lung Cancer. *N. Engl. J. Med.* **2014**, *371*, 2167–2177, doi:10.1056/nejmoa1408440.
11. Solomon, B.J.; Kim, D.-W.; Wu, Y.-L.; Nakagawa, K.; Mekhail, T.; Felip, E.; Cappuzzo, F.; Paolini, J.; Usari, T.; Tang, Y.; et al. Final Overall Survival Analysis From a Study Comparing First-Line Crizotinib Versus Chemotherapy in ALK-Mutation-Positive Non–Small-Cell Lung Cancer. *J. Clin. Oncol.* **2018**, *36*, 2251–2258, doi:10.1200/jco.2017.77.4794.
12. Wu, Y.-L.; Lu, S.; Lu, Y.; Zhou, J.; Shi, Y.-K.; Sriuranpong, V.; Ho, J.; Ong, C.K.; Tsai, C.-M.; Chung, C.-H.; et al. Results of PROFILE 1029, a Phase III Comparison of First-Line Crizotinib versus Chemotherapy in

East Asian Patients with ALK-Positive Advanced Non–Small Cell Lung Cancer. *J. Thorac. Oncol.* **2018**, *13*, 1539–1548, doi:10.1016/j.jtho.2018.06.012.

13. Shan, J.; Xiong, Y.; Wang, D.; Xu, M.; Yang, Y.I.; Gong, K.; Yang, Z.; Wang, G.E.; Yang, X. Nedaplatin-versus cisplatin-based chemotherapy in the survival time of patients with non-small cell lung cancer. *Mol. Clin. Oncol.* **2015**, *3*, 543–549, doi:10.3892/mco.2015.504.
14. Sandler, A.; Gray, R.; Perry, M.C.; Brahmer, J.; Schiller, J.H.; Dowlati, A.; Lilenbaum, R.; Johnson, D.H. Paclitaxel–carboplatin alone or with bevacizumab for non–small-cell lung cancer. *N. Engl. J. Med*. **2006**, *355*, 2542–2550.
15. Comella, P.; Frasci, G.; Panza, N.; Manzione, L.; De Cataldis, G.; Cioffi, R.; Maiorino, L.; Micillo, E.; Lorusso, V.; Di Rienzo, G.; et al. Randomized Trial Comparing Cisplatin, Gemcitabine, and Vinorelbine With Either Cisplatin and Gemcitabine or Cisplatin and Vinorelbine in Advanced Non–Small-Cell Lung Cancer: Interim Analysis of a Phase III Trial of the Southern Italy Cooperative Oncology Group. *J. Clin. Oncol*. **2000**, *18*, 1451–1457, doi:10.1200/jco.2000.18.7.1451.
16. Zhou, C.; Wu, Y.-L.; Chen, G.; Liu, X.; Zhu, Y.; Lu, S.; Feng, J.; He, J.; Han, B.; Wang, J.; et al. BEYOND: A Randomized, Double-Blind, Placebo-Controlled, Multicenter, Phase III Study of First-Line Carboplatin/Paclitaxel Plus Bevacizumab or Placebo in Chinese Patients With Advanced or Recurrent Nonsquamous Non–Small-Cell Lung Cancer. *J. Clin. Oncol.* **2015**, *33*, 2197–2204, doi:10.1200/jco.2014.59.4424.
17. Mok, T.S.K.; Wu, Y.-L.; Kudaba, I.; Kowalski, D.M.; Cho, B.C.; Turna, H.Z.; Castro, G., Jr.; Srimuninnimit, V.; Laktionov, K.K.; Bondarenko, I.; et al. Pembrolizumab versus chemotherapy for previously untreated, PD-L1-expressing, locally advanced or metastatic non-small-cell lung cancer (KEYNOTE-042): A randomised, open-label, controlled, phase 3 trial. *Lancet* **2019**, *393*, 1819–1830, doi:10.1016/s0140-6736(18)32409-7.
18. Reck, M.; Rodríguez-Abreu, D.; Robinson, A.G.; Hui, R.; Csőszi, T.; Fülöp, A.; Gottfried, M.; Peled, N.; Tafreshi, A.; Cuffe, S.; et al. Pembrolizumab versus Chemotherapy for PD-L1–Positive Non–Small-Cell Lung Cancer. *N. Engl. J. Med*. **2016**, *375*, 1823–1833.
19. Reck, M.; Rodríguez–Abreu, D.; Robinson, A.G.; Hui, R.; Csőszi, T.; Fülöp, A.; Gottfried, M.; Peled, N.; Tafreshi, A.; Cuffe, S.; et al. Updated Analysis of KEYNOTE-024: Pembrolizumab Versus Platinum-Based Chemotherapy for Advanced Non–Small-Cell Lung Cancer With PD-L1 Tumor Proportion Score of 50% or Greater. *J. Clin. Oncol.* **2019**, *37*, 537–546, doi:10.1200/jco.18.00149.
20. Herbst, R.S.; Giaccone, G.; De Marinis, F.; Reinmuth, N.; Vergnenegre, A.; Barrios, C.H.; Morise, M.; Felip, E.; Andric, Z.; Geater, S.; et al. Atezolizumab for First-Line Treatment of PD-L1–Selected Patients with NSCLC. *N. Engl. J. Med.* **2020**, *383*, 1328–1339, doi:10.1056/nejmoa1917346.
21. Paz-Ares, L.; Luft, A.; Vicente, D.; Tafreshi, A.; Gümüş, M.; Mazières, J.; Hermes, B.; Çay Şenler, F.; Csőszi, T.; Fülöp, A.; et al. Pembrolizumab plus Chemotherapy for Squamous Non–Small-Cell Lung Cancer. *N. Engl. J. Med.* **2018**, *379*, 2040–2051, doi:10.1056/nejmoa1810865.
22. Socinski, M.A.; Jotte, R.M.; Cappuzzo, F.; Orlandi, F.; Stroyakovskiy, D.; Nogami, N.; Rodríguez-Abreu, D.; Moro-Sibilot, D.; Thomas, C.A.; Barlesi, F.; et al. Atezolizumab for First-Line Treatment of Metastatic Nonsquamous NSCLC. *N. Engl. J. Med.* **2018**, *378*, 2288–2301, doi:10.1056/nejmoa1716948.
